# Supplementary material for: Combining Functional Genomics and Whole-Genome Sequencing to Detect Antibiotic Resistance Genes in Bacterial Strains Co-Occurring Simultaneously in a Brazilian Hospital
Source: Antibiotics (Basel). 2021 Apr 11;10(4):419. doi: 10.3390/antibiotics10040419 (PMC8070361; doi:10.3390/antibiotics10040419)
Supplement: Supplementary file 1 [file antibiotics-10-00419-s001.pdf]

Combining functional genomics and whole genome sequencing to detect antibiotic resistance genes in bacterial strains co-occurring simultaneously in a Brazilian hospital

by

Tiago Cabral Borelli[1$], Gabriel Lencioni Lovate[1$], Ana Flavia Tonelli Scaranello[1], Lucas Ferreira Ribeiro[1], Livia Zaramela[2], Felipe Marcelo Pereira-dos-Santos[3], Rafael Silva-Rocha[3#] and María-Eugenia Guazzaroni[1#]

[1]Department of Biology, Faculdade de Filosofia, Ciências e Letras de Ribeirão Preto, University of São Paulo, São Paulo, SP, Brazil
[2]Department of Pediatrics, University of California San Diego, San Diego, California, USA
[3]Department of Cell and Molecular Biology, Faculdade de Medicina de Ribeirão Preto, University of São Paulo, São Paulo, SP, Brazil

[$]Both authors contributed equally to this work
[#]Both authors contributed equally to this work

*Correspondence to: María-Eugenia Guazzaroni, meguazzaroni@ffclrp.usp.br
Faculdade de Filosofia, Ciências e Letras de Ribeirão Preto, Universidade de São Paulo.
Av. Bandeirantes, 3.900. CEP: 14049-901, Ribeirão Preto, São Paulo, Brazil.

Supporting Materials

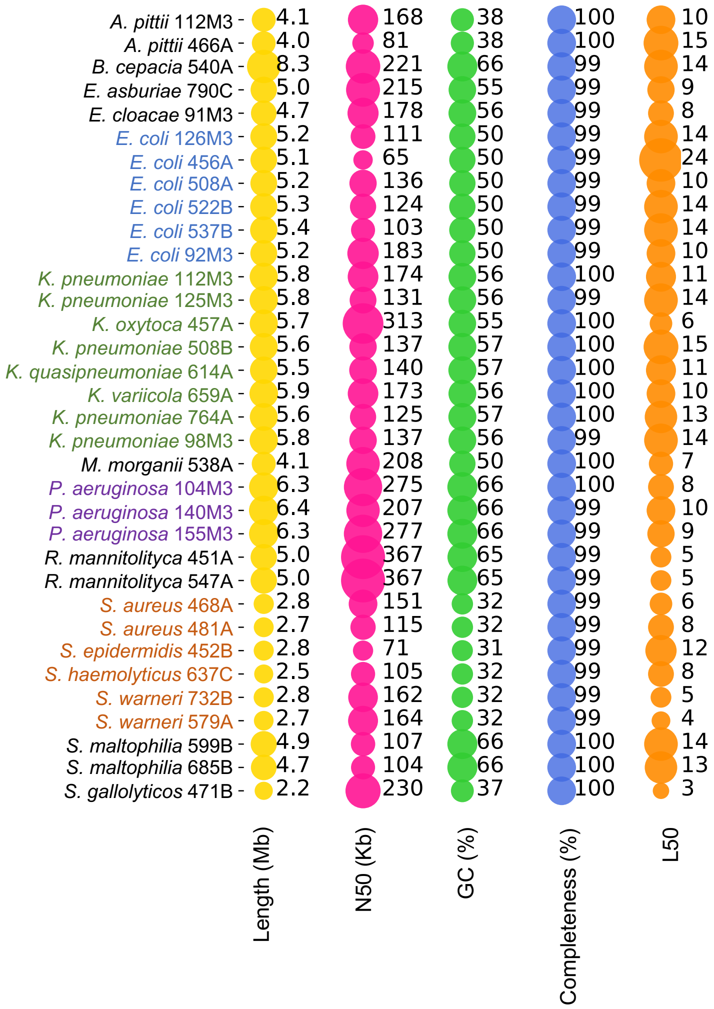

	Length (Mb)	N50 (Kb)	GC (%)	Completeness (%)	L50
A. pittii 112M3	4.1	168	38	100	10
A. pittii 466A	4.0	81	38	100	15
B. cepacia 540A	8.3	221	66	99	14
E. asburiae 790C	5.0	215	55	99	9
E. cloacae 91M3	4.7	178	56	99	8
E. coli 126M3	5.2	111	50	99	14
E. coli 456A	5.1	65	50	99	24
E. coli 508A	5.2	136	50	99	10
E. coli 522B	5.3	124	50	99	14
E. coli 537B	5.4	103	50	99	14
E. coli 92M3	5.2	183	50	99	10
K. pneumoniae 112M3	5.8	174	56	100	11
K. pneumoniae 125M3	5.8	131	56	99	14
K. oxytoca 457A	5.7	313	55	100	6
K. pneumoniae 508B	5.6	137	57	100	15
K. quasipneumoniae 614A	5.5	140	57	100	11
K. variicola 659A	5.9	173	56	100	10
K. pneumoniae 764A	5.6	125	57	100	13
K. pneumoniae 98M3	5.8	137	56	99	14
M. morganii 538A	4.1	208	50	100	7
P. aeruginosa 104M3	6.3	275	66	100	8
P. aeruginosa 140M3	6.4	207	66	99	10
P. aeruginosa 155M3	6.3	277	66	99	9
R. mannitolityca 451A	5.0	367	65	99	5
R. mannitolityca 547A	5.0	367	65	99	5
S. aureus 468A	2.8	151	32	99	6
S. aureus 481A	2.7	115	32	99	8
S. epidermidis 452B	2.8	71	31	99	12
S. haemolyticus 637C	2.5	105	32	99	8
S. warneri 732B	2.8	162	32	99	5
S. warneri 579A	2.7	164	32	99	4
S. maltophilia 599B	4.9	107	66	100	14
S. maltophilia 685B	4.7	104	66	100	13
S. gallolyticos 471B	2.2	230	37	100	3

Figure S1. General features of genomes sequenced in this study.

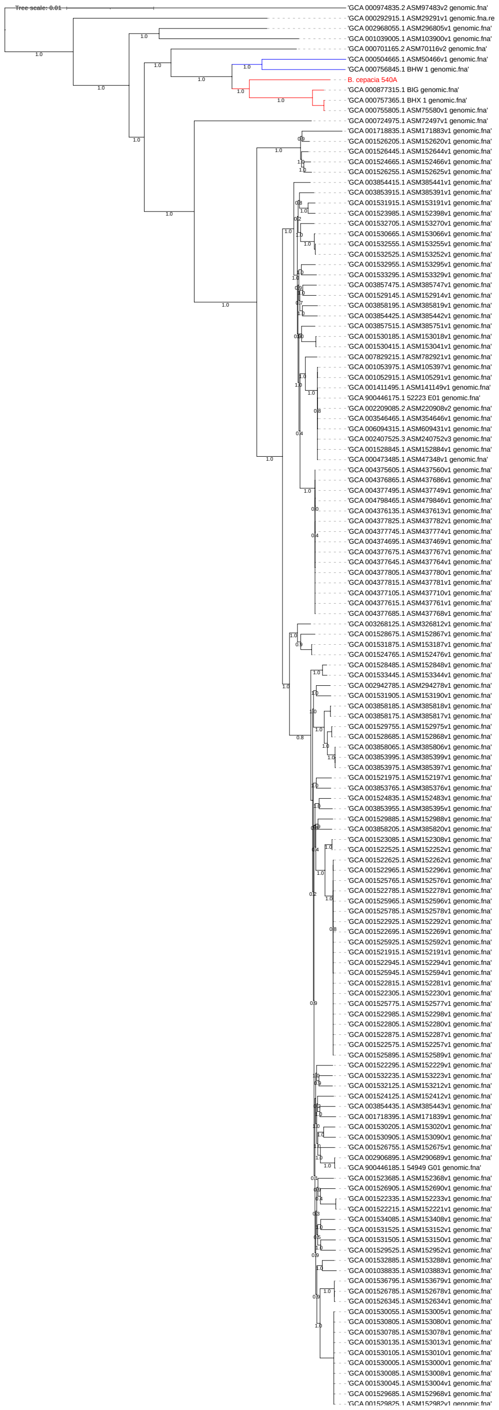

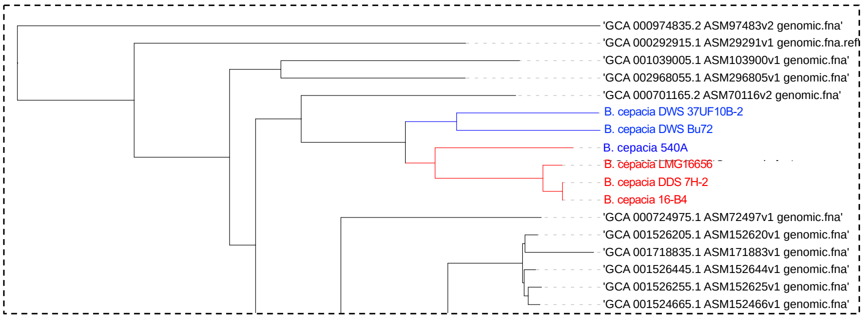

Accession	Strain	Year	Site
GCA_000701165	JKB9	-	Korea, soil
GCA_000756845	DWS 37UF10B-2	2007	Australia, soil
GCA_000504665	Bu72	-	Endophytic bacterium isolated yellow lupine
GCA_000877315	LMG16656	1989	Sputum from cystic fibrosis patient, Edinburgh
GCA_000755805	DDS 7H-2	2005	Australia, aerosol
GCA_000757365	DWS 16B-4	-	Australia, clinical strain

Figure S2. Phylogenetic tree of *B. cepacia* 540A and total number of available *B. cepacia* genomes (166) in NCBI at March 2020. NCBI accession number are indicated for *B. cepacia* strains compared in the figure. Blue branches are two *B. cepacia* endophytic strains isolated in Australia, while red branches are *B. cepacia* isolated from patients. Phylogenomic analyses were performed using Parsnp and Gingr (56) and phylogenetic trees visualized using iTOL (57).

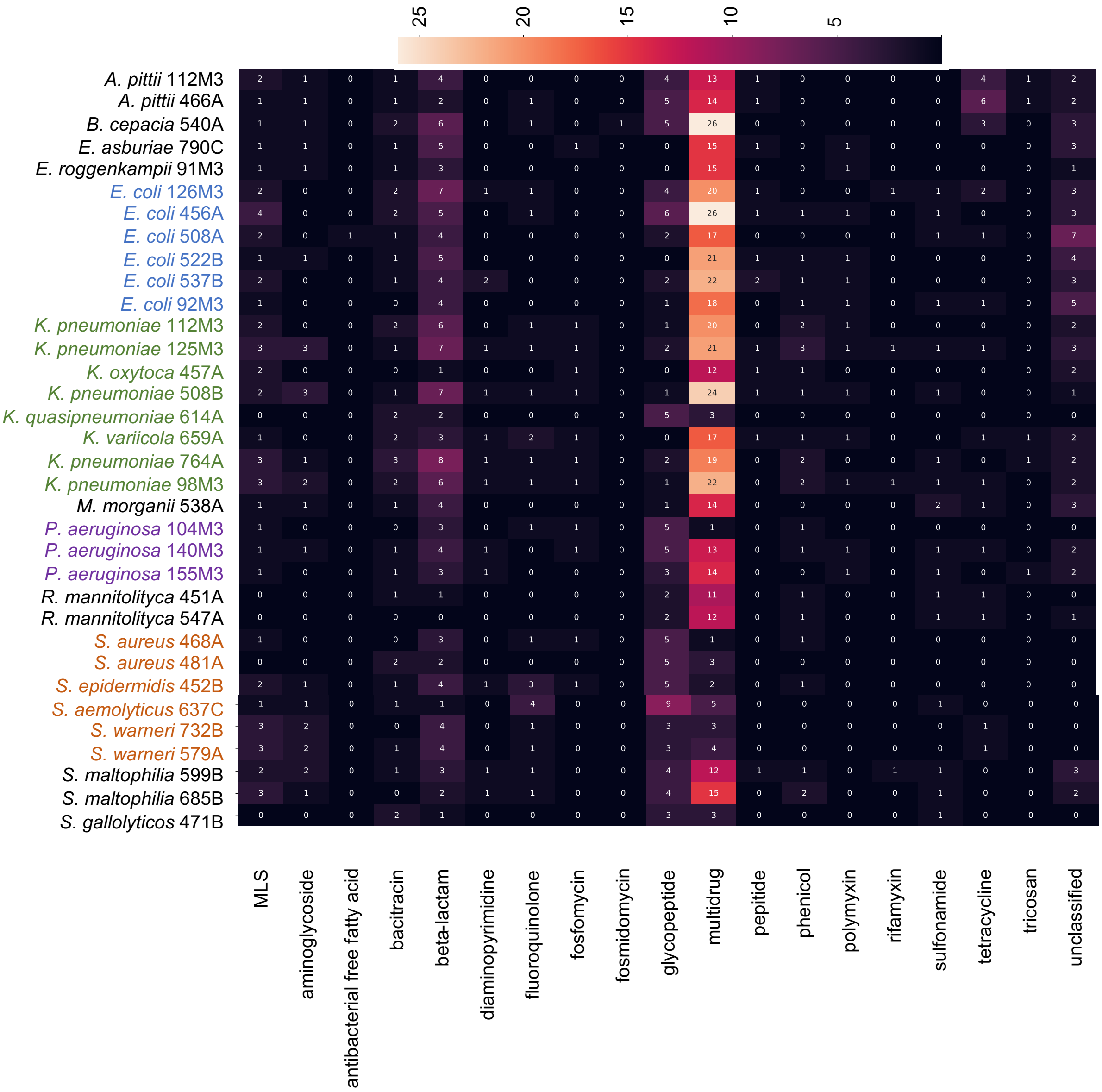

Figure S3. Heatmap showing the presence of ARGs identified by DeepARG in nearly sequenced genomes.

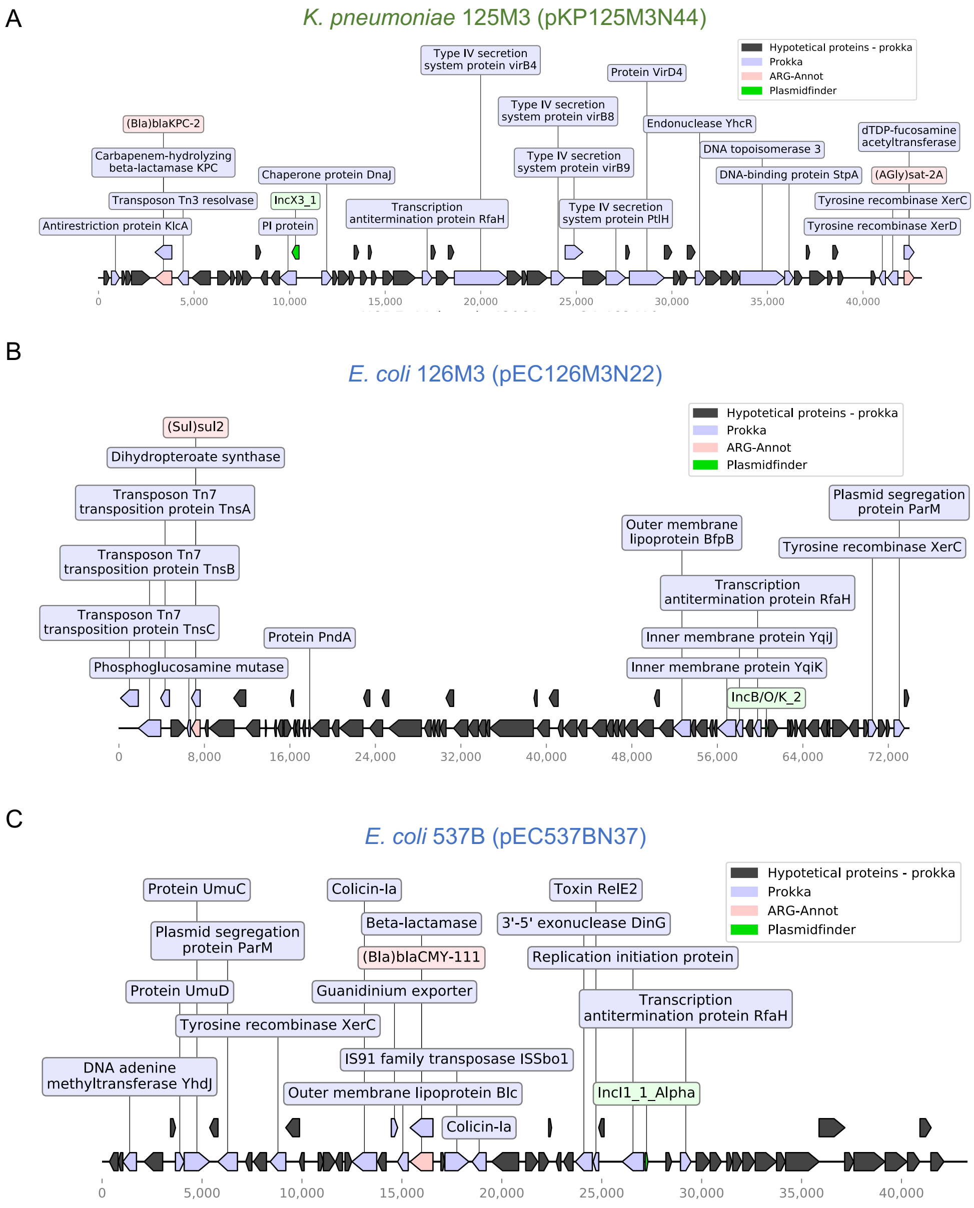

Figure S4. Schematic representation of genes in identified plasmids exposing the coexistence of ARGs with transposon elements. **A**) Plasmid pKP125M3N44 (43 kb) from *K. pneumoniae* 125M3. This plasmid is identical to plasmid pKP98M3N42 from *K. pneumoniae* 98M3 (Fig. 4A). **B**) Plasmid pEC126M3N22 (73,9 kb) from *E. coli 126M3*. **C**) Plasmid pEC537BN37 (43,3 kb) from *E. coli 537B*. Legends represents the colors code for the identified genes.

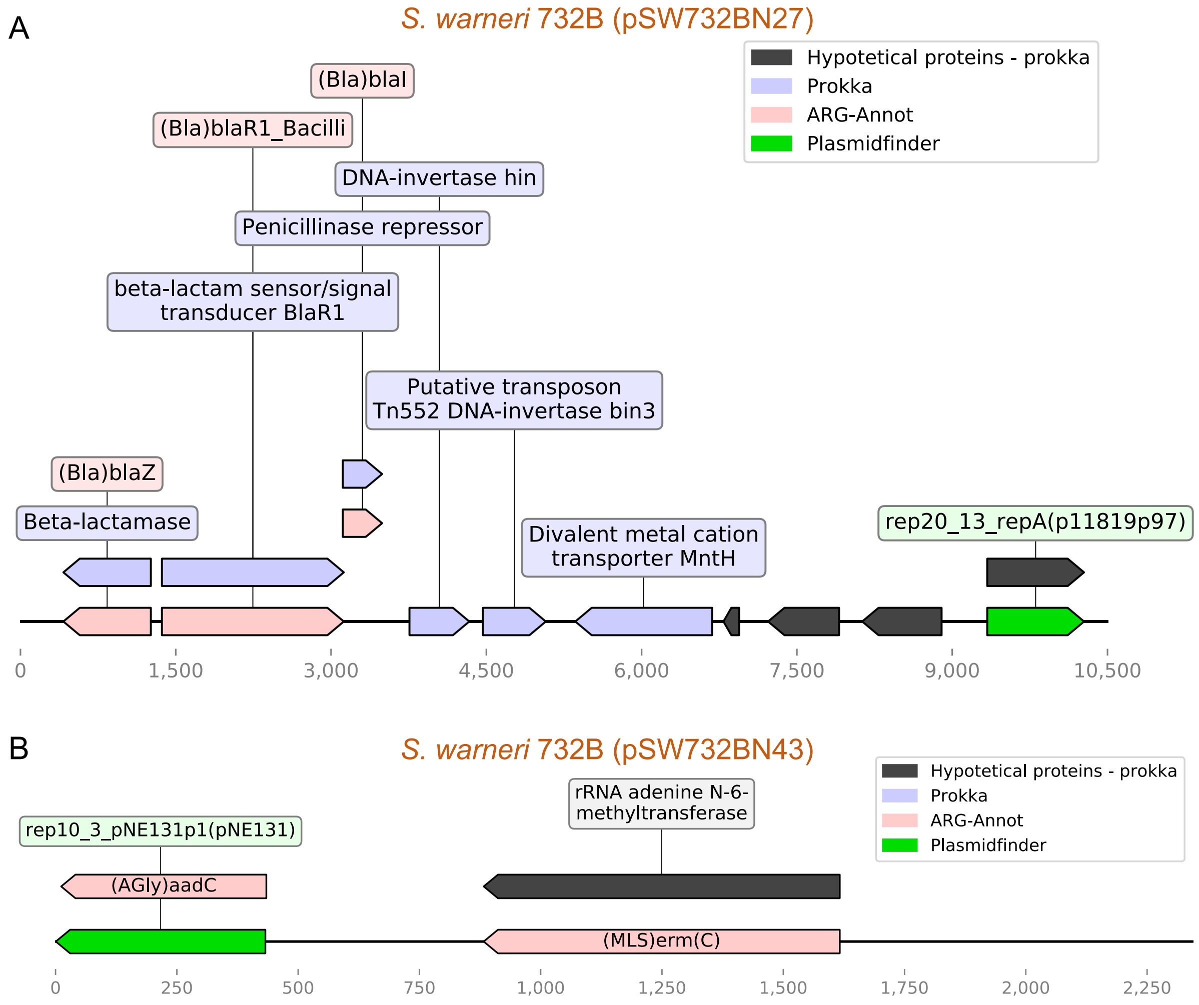

Figure S5. Schematic representation of plasmids identified in *Staphylococcus* strains showing the coexistence of ARGs with transposon elements. **A**) Plasmid pSW732BN27 (10.5 kb) from *S. warneri 732B*. **B**) Plasmid pSW732BN43 (~2.3kb) from *S. warneri 732B*.

Table S1 - Comparison between ARG-ANNOT and susceptibly tests.

Species	Isolate	ARG	Origin	Predicted resistance	Phenotype (resistant to)
E. coli	126M3	*bla*$_{OXA-1}$	Acquired	Cephalosporin	Ceftazidime<br>Cefotaxime<br>Cefepime
				Penam (penicillin)	Ampicillin
		bla$_{KPC-2}$	Acquired	Carbapenem	Ertapenem<br>Meropenem
				Cephalosporin	Cefotaxime<br>Ceftazidime<br>Cefepime
				Monobactam	Not tested

				Penam (penicillin)	Ampicillin
	522B	$\mathit{bla}_{\text{CTX-M-15}}$	Acquired	Cephalosporin	Cefotaxime<br>Ceftazidime<br>Cefepime
	537B	$\mathit{bla}_{\text{CMY-111}}$	N (*Citrobacter freundii*)	Cephamycin	Cefoxitin
	92M3	$\mathit{bla}_{\text{TEM-105}}$	Acquired	Cephalosporin	Susceptible
				Monobactam	Not tested
				Penam (penicillin)	Ampicillin
				Penem	Not tested
	508A	$\mathit{bla}_{\text{OXA-1}}$	Acquired	Cephalosporin	Susceptible
				Penam (penicillin)	Ampicillin
K. pneumoniae	112M3	$\mathit{bla}_{\text{SHV-187}}$	Natural (*K. pneumoniae*)	Carbapenem	Ertapenem<br>Meropenem
				Cephalosporin	Cefotaxime<br>Ceftazidime<br>Cefepime
				Penam (penicillin)	Ampicillin
	98M3	$\mathit{bla}_{\text{SHV-11}}$	Acquired	Carbapenem	Ertapenem<br>Meropenem
				Cephalosporin	Cefotaxime<br>Ceftazidime<br>Cefepime
				Penam (penicillin)	Ampicillin
		$\mathit{bla}_{\text{KPC-2}}$	Acquired	Carbapenem	Ertapenem<br>Meropenem
				Cephalosporin	Cefotaxime<br>Ceftazidime<br>Cefepime
				Monobactam	Not tested
				Penam (penicillin)	Ampicillin
		$\mathit{bla}_{\text{CTX-M-19}}$	Acquired	Cephalosporin	Cefotaxime<br>Ceftazidime<br>Cefepime
		$\mathit{bla}_{\text{TEM-105}}$	Acquired	Cephalosporin	Cefotaxime<br>Ceftazidime<br>Cefepime
				Monobactam	Not tested
				Penam (penicillin)	Ampicillin
				Penem	Not tested
	764A	$\mathit{bla}_{\text{SHV-106}}$	Natural (*Enterobacter sp.* MGH 14)	Cephalosporin	Cefotaxime<br>Ceftazidime<br>Cefepime

				Carbapenem	Ertapenem<br>Meropenem
				Penam (penicillin)	Ampicillin
		$bla_{TEM-105}$	Acquired	Cephalosporin	Cefotaxime<br>Ceftazidime<br>Cefepime
				Monobactam	Not tested
				Penam (penicillin)	Ampicillin
				Penem	Not tested
		$bla_{CTX-M-15}$	Acquired	Cephalosporin	Cefotaxime<br>Ceftazidime<br>Cefepime
		bla_{OXA-1}	Acquired	Cephalosporin	Cefotaxime<br>Ceftazidime<br>Cefepime
				Penam (penicillin)	Ampicillin
	508B	bla_{SHV-11}	Acquired	Carbapenem	Susceptible
				Cephalosporin	Cefotaxime<br>Ceftazidime<br>Cefepime
				Penam (penicillin)	Ampicillin
		$bla_{CTX-M-15}$	Acquired	Cephalosporin	Cefotaxime<br>Ceftazidime<br>Cefepime
		bla_{OXA-1}	Acquired	Cephalosporin	Cefotaxime<br>Ceftazidime<br>Cefepime
				Penam (penicillin)	Ampicillin
		bla_{LAP-2}	Acquired	Aminoglycoside<br>Fluoroquinolone<br>Rifamycin<br>Tetracycline	Gentamicin<br>Ciprofloxacin<br>Not tested<br>Tigercycline
	125M3	bla_{SHV-11}	Acquired	Carbapenem	Ertapenem<br>Meropenem
				Cephalosporin	Cefotaxime<br>Ceftazidime<br>Cefepime
				Penam (penicillin)	Ampicillin
		bla_{KPC-2}	Acquired	Carbapenem	Ertapenem<br>Meropenem
				Cephalosporin	Cefotaxime<br>Ceftazidime<br>Cefepime
				Monobactam	Not tested

				Penam (penicillin)	Ampicillin
		bla$_{\text{CTX-M-14}}$	Natural (*Kluyvera sp.* N03-0458)	Cephalosporin	Cefotaxime<br>Ceftazidime<br>Cefepime
		bla$_{\text{TEM-105}}$	Acquired	Cephalosporin<br>Monobactam<br>Penam (penicillin)<br>Penem	Cefotaxime<br>Ceftazidime<br>Cefepime<br>Not tested<br>Ampicillin<br>Not tested
K. variicola	659A	*bla*$_{\text{LEN-24}}$	Natural (*K. pneumoniae*)	Penem<br>Penam (penicillin)	Not tested<br>Ampicillin
K. quasipneumoniae	614A	*bla*$_{\text{OKP-B-9}}$	Natural (*K. pneumoniae*)	Cephalosporin<br>Penam (penicillin)	Susceptible<br>Ampicillin
K. oxytoca	457A	*bla*$_{\text{OXY2-1}}$	Natural (*K. oxytoca*)	Cephalosporin<br>Monobactam<br>Penam (penicillin)	Cefotaxime<br>Ceftazidime<br>Cefepime<br>Not tested<br>Ampicillin

Table S2 – Phage-related contigs identified by Phaster.

Strain	Contig	Completeness Score*	Start-End
K. pneumoniae 508B	NODE_53_length_23265_cov_16.832643	Questionable (75)	9692-22726
E. coli 537B	NODE_52_length_27715_cov_8.380540	Intact (100)	703-9465
K. pneumoniae 98M3	NODE_52_length_23307_cov_9.564590	Questionable (75)	398-12901
K. pneumoniae 125M3	NODE_57_length_13879_cov_8.456749	Incomplete (30)	4970-13790

*The criteria for completeness scoring are based on the number of phases CDSs found in the phage region

Table S3 – Replicons identified by bacWSGTdb for *K. pneumonia* 508B and 98M3 genomes.

Strain	Replicon	Contig	Identity (%)	Start-End
K. pneumoniae 508B	IncC	NODE_15_length_136795_cov_22.099162*	100	28286 - 28702
	IncFIB(K)	NODE_66_length_8337_cov_24.095654	100	3603 - 4162
	Col440I	NODE_68_length_5245_cov_640.966187	93.64	286 - 395
	Col440II	NODE_68_length_5245_cov_640.966187	90.75	4599 - 4879
	Col440I	NODE_71_length_4632_cov_690.022060	97.37	4298 - 4411
	Col440I	NODE_72_length_4287_cov_589.476839	96.49	1426 - 1539
K. pneumoniae 98M3	IncFII(K)	NODE_27_length_87380_cov_12.014012	100	44394 - 44541
	IncC	NODE_39_length_51018_cov_14.408885	100	21125 - 21541
	IncX3	NODE_42_length_42978_cov_16.990438*	99.73	10037 - 10410
	ColRNAI	NODE_62_length_9393_cov_64.187218	100	4306 - 10410
	IncFIB(K)	NODE_67_length_6761_cov_15.677875	100	2698 - 3257

*Contigs with co-occurrence of antibiotic resistance genes

Table S4 – Most similar strains* identified by bacWSGTdb SNP strategy for *K. pneumonia* 98M3.

**Isolate**	**Accession number**	**Country**	**Collection Year**
HSP08	SBDB01	Brazil	2016
P72	SBDF01	Brazil	2015
HSP31	SBCZ01	Brazil	2016
P73	SBDE01	Brazil	2016
P27	SBDL01	Brazil	2015
P43	SBDI01	Brazil	2015
P39	SBDJ01	Brazil	2014
P38	SBDK01	Brazil	2016
P88	SBDC01	Brazil	2015
HSP15	SBDD01	Brazil	2016
HSP16	SBDA01	Brazil	2016
P48	SBDH01	Brazil	2016
P71	SBDG01	Brazil	2016
T38_P39_mcr1_tc	VIAI01	Thailand	2018
TUM16641	BFCA01	Japan	-
1849	MZZT01	Brazil	2013

*Only isolates with country information were included in the analysis.

Table S5 – Most similar strains* identified by bacWSGTdb SNP strategy for *K. pneumonia* 508B.

**Isolate**	**Accession number**	**Country**	**Collection Year**
k1534	FLCC01	United Kingdom	2007
k2037	FLFE01	United Kingdom	2009
k557	FLDM01	United Kingdom	2003
EuSCAPE_AT004	UKBH01	Austria	2014
EuSCAPE_CZ034	UKFW01	Czech Republic	2014
EuSCAPE_AT032	UKBF01	Austria	2013
EuSCAPE_CZ032	UKFP01	Czech Republic	2013

*Only isolates with country information were included in the analysis.
